# A proof-of-concept study on CGRP plasma levels of migraineurs during a 6-month treatment with ERENUMAB

**DOI:** 10.1186/s10194-020-01193-4

**Published:** 2020-10-21

**Authors:** Giuseppe Tringali, Catello Vollono, Paolo Calabresi, Pierluigi Navarra

**Affiliations:** 1 Department of Healthcare Surveillance and Bioethics, Section of Pharmacology, Università Cattolica del Sacro Cuore, Largo F. Vito 1, 00168 Rome, Italy; 2 Neurofisiopatologia - Fondazione Policlinico Universitario Agostino Gemelli IRCCS, Rome, Italy; 3 Neurologia - Università Cattolica del Sacro Cuore, Rome, Italy; 4 Neurologia - Fondazione Policlinico Universitario Agostino Gemelli IRCCS, Rome, Italy; 5 Pharmacology - Fondazione Policlinico Universitario Agostino Gemelli IRCCS, Rome, Italy

**Keywords:** CGRP, CGRP receptor, Monoclonal antibodies, Erenumab, Migraine

## Abstract

The introduction of monoclonal antibodies (mAbs) against calcitonin-gene related peptide (CGRP) or CGRP receptors in the treatment of migraine raised concerns on the possible risks associated to the long-term inhibition of CGRP physiological functions. In this proof-of-concept study, we have measured the circulating levels of CGRP in 7 patients with high-frequency episodic migraine receiving the anti-CGRP receptor mAb erenumab for at least 6 months, to test the hypothesis that long-term blockade of CGRP receptors induces an increase in systemic CGRP levels via a classical up-regulation mechanism.

Plasma CGRP levels were measured by a validated radioimmunoassay at baseline, and after 1 and 6 months of treatment with erenumab, 70 mg given sc every 4 weeks.

We found (data expressed as the means ± SD): 38.34 ± 30.74 pg CGRP/ml of plasma at baseline, 38.19 ± 29.23 pg/ml after 1 month and 53.89 ± 28.03 pg/ml after 6 months of treatment. Thus, the average increase in plasma CGRP levels after 6 months of treatment was about + 40% compared to both baseline and 1-month treatments; such difference was not statistically significant because of high SD values in all groups.

These preliminary findings need to be confirmed in larger, sufficiently powered experiments.

In the June 2019 issue, the journal *Peptides* published our last work on calcitonin gene-related peptide (CGRP), a review dealing with the anti-CGRP and anti-CGRP receptor monoclonal antibodies (mAbs) recently introduced for migraine treatment [1]. The paper addressed the concerns raised by the potential risks ensuing a long-term inhibition of CGRP functions, and we discussed whether the different action mechanisms of these mAbs (i.e. quenching systemic CGRP vs blocking its receptors) might be associated to different safety profiles. Among other issues, we made a point of measuring plasma CGRP levels during long-term treatments with anti-CGRP mABs [1]. Concerns about the potential risks associated to long-term blockade of CGRP or CGRP receptor are shared by several groups [2–5], whereas a more optimistic view foresees a remarkable safety profile, based on the concept that anti-CGRP and anti-CGRP receptor mAbs knockdown, but do not knockout CGRP signaling [6].

First-in-class mAb, the anti-CGRP receptor erenumab obtained a marketing authorization in the European Union on July 26, 2018 (https://www.ema.europa.eu/en/medicines/human/EPAR/aimovig), but only very recently the price negotiation process has been completed in Italy (https://www.gazzettaufficiale.it/eli/gu/2020/07/21/182/sg/pdf). During the time that erenumab was not commercially available, the drug has been provided through an expanded access program, and a limited number of patients have been treated at our Headache Clinics, ‘*Fondazione Policlinico Gemelli IRCCS*’ Academic Hospital in Rome.

Having in mind the risk hypotheses postulated in our review [1], we used a previously validated CGRP radioimmunoassay [7] to measure the levels of circulating free CGRP in the plasma of patients included in the expanded access program of our Center, and treated with erenumab at approved dosages for at least 6 months. Seven patients with high-frequency episodic migraine, meeting the criteria of erenumab labeling, had a complete set of blood samples collected: all the patients received the 70-mg dose. All patients provided an informed consent for use of biological samples, according to the rules of Gemelli Hospital, and the study protocol was approved by the Independent Ethics Committee of the hospital.

We found (data expressed as pg CGRP/ml of plasma, the means ± SD of 7 replicates per group): 38.34 ± 30.74 at baseline; 38.19 ± 29.23 after 1 month of treatment (i.e. after a single administration); 53.89 ± 28.03 after 6 months of treatment (i.e. at steady-state). Thus, the average increase in circulating CGRP after 6 months of erenumab treatment was about + 40% compared to both baseline and 1-month treatments, although such difference was not statistically significant because of huge SDs in all groups.

We are fully aware of the limitations of this study. The number of patients included is small, and overall variability is subsequently high. Assuming a SD around 30 pg/ml, for a size effect of + 40% to be statistically significant, a sample size of at 55 subject is required, with a power of 80% and a risk of type-I error < 5% in a two-tail test. Nonetheless, we believe it may be useful to publish this proof-of-concept study, in order to stimulate discussion on the issue of the biological effects of this new class of drugs, and possibly to prompt further, adequately powered studies on this topic. Such patho-physiological investigations might usefully complement the clinical studies that are currently under way to establish the effectiveness of erenumab in the real-world setting [8].

## Data Availability

Raw data are available upon request to the Corresponding author.
